# Recurrent Cholestatic Jaundice in a Young Indian Adult: A Clinical Diagnosis of Benign Recurrent Intrahepatic Cholestasis

**DOI:** 10.7759/cureus.113970

**Published:** 2026-08-04

**Authors:** Sudhanshu Sekhar Panda, Sibabrata Panda, Aparupa Jujharsingh, Vyomakesh Patra

**Affiliations:** 1 General Medicine, Maharaja Krishna Chandra Gajapati Medical College and Hospital, Berhampur, IND

**Keywords:** benign recurrent intrahepatic cholestasis, low-normal ggt cholestasis, pruritus, recurrent jaundice, ursodeoxycholic acid

## Abstract

Benign recurrent intrahepatic cholestasis (BRIC) is a rare hereditary cholestatic disorder characterized by recurrent episodes of intermittent cholestatic jaundice and pruritus without causing detectable lasting liver damage. Owing to its rarity and the absence of pathognomonic findings, the diagnosis is often delayed and requires exclusion of more common causes of recurrent cholestasis. We report the case of a 25-year-old male who presented with recurrent episodes of severe cholestatic jaundice with intense generalized pruritus. The patient clinically and biochemically recovered after treatment with ursodeoxycholic acid (UDCA). Based on the characteristic recurrence pattern, severe pruritus, normal serum gamma-glutamyl transferase (GGT) levels during the current episode, normal hepatobiliary imaging, complete clinical recovery between attacks, and exclusion of alternative causes of cholestasis, a presumptive clinical diagnosis of BRIC was made. Although genetic confirmation was not done, the clinical features were most consistent with BRIC.

## Introduction

Benign recurrent intrahepatic cholestasis (BRIC) is a rare hereditary cholestatic liver disorder characterized by recurrent episodes of intrahepatic cholestasis with symptom-free intervals and complete clinical and biochemical recovery. It is an exceptionally rare inherited cholestatic disorder, with an estimated prevalence of approximately one per 50,000-100,000 individuals. It was first described by Summerskill and Walshe in 1959 [1]. Benign recurrent intrahepatic cholestasis is classified into two major subtypes: BRIC type 1, caused by mutations in the ATP8B1 gene, and BRIC type 2, caused by mutations in the ABCB11 gene. These genetic abnormalities impair canalicular bile secretion and lead to episodic cholestasis without progressive destruction of hepatic architecture in most affected individuals [1-3]. Both BRIC and progressive familial intrahepatic cholestasis (PFIC) are allelic disorders caused by mutations in the same canalicular bile transport genes, primarily ATP8B1 and ABCB11. Benign recurrent intrahepatic cholestasis represents the milder end of the disease spectrum and is characterized by recurrent episodes of cholestasis with complete recovery between attacks, whereas PFIC is the progressive phenotype that may lead to fibrosis, cirrhosis, and liver failure [2-5].

Diagnosis remains difficult because no single clinical, biochemical, radiological, or histopathological feature is pathognomonic. Instead, the diagnosis relies on recognition of a characteristic clinical pattern together with exclusion of alternative causes of recurrent cholestasis. Laboratory investigations usually demonstrate marked conjugated hyperbilirubinemia and elevated alkaline phosphatase levels with only mild elevations of serum aminotransferases. One of the most useful diagnostic clues is the presence of normal or low serum gamma-glutamyl transferase (GGT) levels despite significant cholestasis [2,3,6]. This reflects impaired canalicular bile salt transport without significant injury to biliary epithelial cells, distinguishing BRIC from cholangiopathies in which GGT levels are typically elevated. Here, we report the case of a 25-year-old male presenting with recurrent episodes of severe cholestatic jaundice associated with intense pruritus and normal serum GGT levels during the documented episode.

## Case presentation

A 25-year-old male presented with yellowish discoloration of the sclera and skin, severe generalized pruritus, dark urine, and pale stool for one month. There was no history of fever, abdominal pain, vomiting, gastrointestinal bleeding, weight loss, alcohol consumption, recent travel, blood transfusion, high-risk sexual behavior, drug intake, herbal medication use, or exposure to hepatotoxic drugs. There was no family history of recurrent jaundice, chronic liver disease, or known inherited liver disorders. The patient's parents were non-consanguineous. Although a negative family history does not exclude autosomal recessive disorders such as BRIC, no clinical or biochemical features suggestive of alternative hereditary jaundice syndromes were identified. The patient had a past history of similar symptoms one year back, lasting for one month and resolving spontaneously with conservative management. Laboratory investigation reports of the first episode were not available with the patient. Therefore, biochemical comparison between the two episodes was not possible.

On examination, the patient was conscious and oriented to time, place, and person with stable vitals. He had profound icterus and excoriation marks on his limbs, back, and trunk. There was no cyanosis, clubbing, pedal edema, or lymphadenopathy. No signs of organomegaly, portal hypertension, or chronic liver disease. Neurological examination was normal with the absence of Kayser-Fleischer rings on slit-lamp examination.

Laboratory investigations showed cholestasis with a total bilirubin of 28.5 mg/dL, direct bilirubin of 19.7 mg/dL, alkaline phosphatase (ALP, 842 U/L), aspartate aminotransferase (AST, 62 U/L), and alanine aminotransferase (ALT, 58 U/L). Serum GGT (32.5 IU/L) remained within normal limits. Viral markers for hepatitis A, B, C, E, and HIV 1 and 2 were negative. The autoimmune liver disease workup, including antinuclear antibody (ANA), anti-smooth muscle antibody (ASMA), anti-mitochondrial antibody (AMA), and anti-liver kidney microsomal antibody (anti-LKM), was unremarkable. Evaluation for Wilson disease, including serum ceruloplasmin and 24-hour urinary copper excretion, was normal (Table 1).

**Table 1:** Laboratory reports of current episode ALT: Alanine transaminase; ALP: Alkaline phosphatase; ANA: Anti-nuclear antibodies; AMA: Anti-mitochondrial antibodies; ASMA: Anti-smooth muscle antibody; AST: Aspartate transaminase; GGT: Gamma-glutamyl transferase; HAV: Hepatitis A virus; HBsAg: Hepatitis B surface antigen; HCV: Hepatitis C virus; HEV: Hepatitis E virus; INR: International normalized ratio; LDH: Lactate dehydrogenase; LKM: Liver kidney microsome

Parameter	Current episode	Reference range
Hemoglobin (g/dL)	12.0	11.0-16.0
Platelet count (x10^3^/mm^3^)	481	150-400
Total leukocyte count (TLC) (cells/mm^3^)	8130	4000-11000
Total bilirubin (mg/dL)	28.5	0.2-1.0
Direct bilirubin (mg/dL)	19.7	0-0.2
Indirect bilirubin (mg/dL)	8.8	0.2-0.8
ALT (IU/L)	58	<45
AST (IU/L)	62	<35
ALP (IU/L)	842	58-306
GGT (U/L)	32.5	10-45
LDH (U/l)	548	225-450
Anti-HAV IgM	Negative	
Anti-HEV IgM	Negative	
HBsAg	Negative	
Anti-HCV	Negative	
HIV-I/II	Non-reactive	
Ceruloplasmin (g/L)	0.25	0.22-0.58
24-h urinary copper excretion (mcg/24 h)	37.4	15-60
Prothrombin time (seconds)	11.5	11-13
Activated partial thromboplastin time (aPTT) (seconds)	27.9	21-26
INR	0.96	
Serum protein (g/dL)	7.4	6.0-8.0
Serum albumin (g/dL)	3.6	3.5-5.2
ANA	Negative	
ASMA	Negative	
LKM-1	Negative	
AMA	Negative	

**Figure 1:**
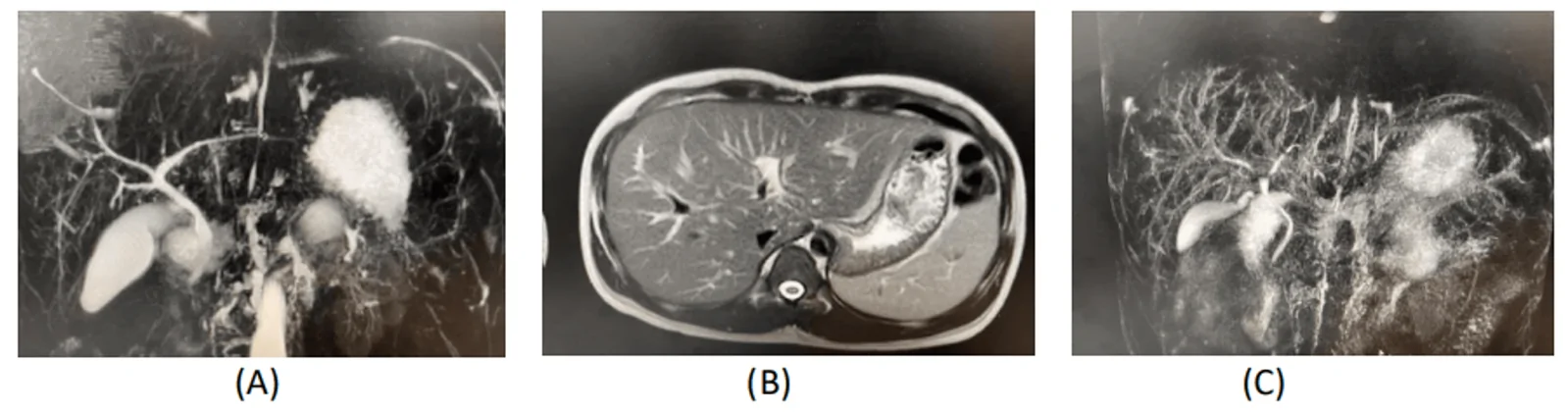
Magnetic resonance cholangiopancreatography imaging A: Normal gallbladder with no calculi or filling defect; B: No intrahepatic biliary dilatation; C: Normal common bile duct

Abdominal ultrasonography showed normal liver size and echotexture without any gallstones or biliary ductal dilatations. Magnetic resonance cholangiopancreatography (MRCP) demonstrated normal liver morphology and normal intrahepatic and extrahepatic biliary channels without any evidence of biliary obstruction, choledocholithiasis, biliary strictures, or space-occupying lesions (Figure 1). With the exclusion of common autoimmune and metabolic causes, a predominantly conjugated familial hyperbilirubinemia was suspected.

**Figure 2:**
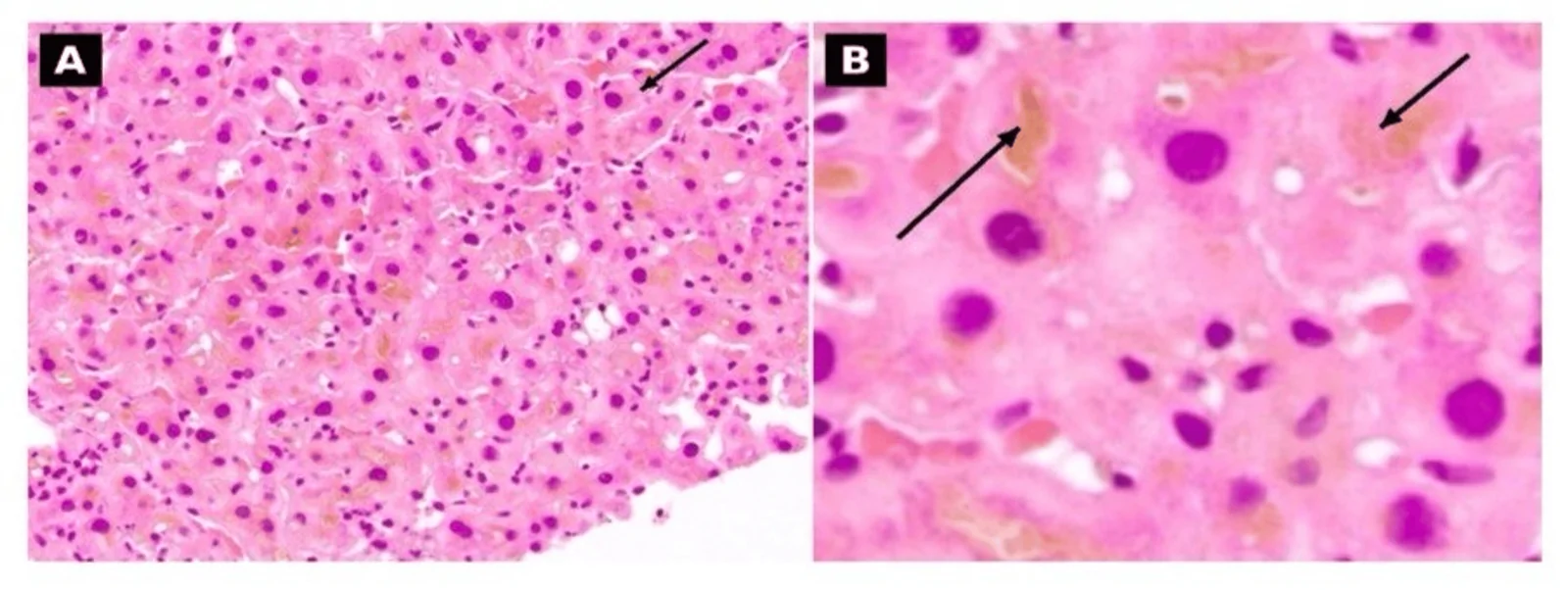
Liver biopsy A: Normal hepatic parenchyma without any Inflammation or fibrosis (black arrow); B: Intrahepatic cholestasis (black arrows) on H&E staining

The liver biopsy showed preserved hepatic lobular architecture and intracytoplasmic and focal canalicular accumulation of golden brown pigment, indicating intrahepatic cholestasis. No features suggestive of autoimmune hepatitis, primary sclerosing cholangitis, or metabolic liver diseases. The combination of bland cholestasis with preserved nonfibrotic parenchyma and absence of significant hepatocellular injury or portal inflammation was consistent with intrahepatic cholestasis without progressive liver damage (Figure 2).

In view of recurrent episodes of severe cholestatic jaundice associated with intense pruritus, normal GGT level during the current episode, normal hepatobiliary imaging, complete recovery between attacks, and exclusion of common causes of cholestasis, a clinical diagnosis of BRIC was established [2]. Genetic testing was not done due to resource limitations.

The patient was managed conservatively with supportive care, including adequate hydration and nutritional support. He was started on ursodeoxycholic acid (UDCA) (15 mg/kg/day) along with antihistamines. Cholestyramine (4 g twice daily) was added because pruritus persisted despite UDCA therapy. The drug was continued for four weeks, resulting in gradual improvement in pruritus, and was discontinued after complete symptomatic resolution. By six weeks, liver biochemistry had returned to normal. The serial biochemical response to treatment is summarized in Table 2. The patient and the family members were advised to have regular follow-ups and counselled about the benign but recurrent nature of the condition.

**Table 2:** Follow-up liver biochemistry ALT: Alanine transaminase; ALP: Alkaline phosphatase; AST: Aspartate transaminase; GGT: Gamma-glutamyl transferase

Parameter	Current admission	Week four	Week six	Reference range
Total bilirubin (mg/dL)	28.5	11.4	0.9	0.2–1.0
Direct bilirubin (mg/dL)	19.7	9.0	0.1	0–0.2
Indirect bilirubin (mg/dL)	8.8	2.4	0.8	0.2-0.8
AST (U/L)	58	36	22	<45
ALT (U/L)	62	29	18	<35
ALP (U/L)	842	281	264	58-306
GGT (U/L)	32.5	31	29	10-45

## Discussion

Benign recurrent intrahepatic cholestasis is a rare inherited cholestatic disorder characterized by recurrent episodes of severe intrahepatic cholestasis with symptom-free intervals and complete clinical and biochemical recovery. The disorder is classified into BRIC type 1 and BRIC type 2, resulting from mutations in the ATP8B1 and ABCB11 genes, respectively [2]. Patients typically present during childhood, adolescence, or early adulthood with recurrent attacks of jaundice and severe pruritus. Other symptoms may include dark urine, pale stools, anorexia, fatigue, malaise, and weight loss. Patients generally remain asymptomatic, and the liver biochemistry is normal between attacks. 

The differential diagnosis of recurrent cholestasis with normal serum GGT includes inherited bile transport disorders such as BRIC and PFIC, as well as drug-induced cholestasis, particularly anabolic steroid-induced cholestasis. In our patient, there was no history of prescription medications, herbal preparations, or anabolic steroid use. Viral, autoimmune, and metabolic liver diseases were excluded by appropriate investigations, while ultrasonography and MRCP demonstrated no biliary obstruction. Liver biopsy showed bland intrahepatic cholestasis without fibrosis or chronic cholangiopathy. Conversely, disorders such as choledocholithiasis, primary sclerosing cholangitis, cholangiocarcinoma, biliary strictures, and IgG4-related cholangitis are typically associated with elevated GGT levels and characteristic imaging abnormalities. Collectively, these findings favored BRIC.

Although genetic testing was not performed because of resource limitations, numerous published cases of BRIC have been diagnosed clinically after exclusion of alternative causes without molecular confirmation [2,7,8]. Our patient fulfilled the clinical criteria proposed by Luketic and Shiffman, including recurrent cholestatic episodes, severe pruritus, normal serum GGT levels during the documented episode, normal biliary imaging, and exclusion of alternative causes. Nevertheless, because BRIC and PFIC represent a recognized phenotypic continuum, an early or mild PFIC phenotype cannot be completely excluded in the absence of molecular confirmation [2].

Benign recurrent intrahepatic cholestasis is a self-limited disease without any residual damage or progression to fibrosis. Treatment is mostly conservative and aimed at symptom control with shortening of episodes. Therapeutic options include UDCA, antihistamines, cholestyramine, and rifampicin [9]. Plasmapheresis and molecular adsorbent recirculating systems (MARS) have been described in refractory cases with severe pruritus [6,7,10,11]. Although BRIC is a benign disease, it may rarely progress to PFIC, which may cause chronic liver disease and cirrhosis. Early diagnosis and prompt management can improve patient outcomes and prevent unnecessary diagnostic interventions.

## Conclusions

This case illustrates a presumptive clinical diagnosis of BRIC in a young adult presenting with recurrent cholestatic jaundice, severe pruritus, normal serum GGT levels during the documented episode, normal biliary imaging, and exclusion of alternative causes. Although molecular confirmation was not done and a mild PFIC phenotype cannot be completely excluded, the overall clinical presentation was most consistent with BRIC. Clinicians should consider BRIC in patients presenting with recurrent low- or normal-GGT cholestasis after exclusion of common causes. Genetic confirmation and longer-term follow-up would further strengthen diagnostic certainty.
